# Assessment of environmental and health risks of potentially toxic elements associated with desert dust particles affected by industrial activities in Isfahan metropolitan

**DOI:** 10.1038/s41598-024-74153-6

**Published:** 2024-10-01

**Authors:** Moslem Yazdani, Hamidreza Karimzadeh, Hamidreza Azimzadeh, Mohsen Soleimani

**Affiliations:** 1 Department of Natural Resources, Isfahan University of Technology, Isfahan, 84156–83111 Iran; 2 Environmental Sciences Department, School of Natural Resources and Desert Studies, Yazd University, Yazd, 89158-18411 Iran

**Keywords:** Particulate matters (PMs), Trace elements, Sandstorms, Health and ecological risks, Desertification, Environmental chemistry, Natural hazards, Risk factors

## Abstract

Dust particles and their associated compounds can adversely affect human health and ecosystems. The aim of this study was to investigate the concentration, health, and ecological risks of selected potentially toxic elements (e.g. Pb, Cd, Cr, Co, Cu, Zn, V, Ni, and As) bound to air particles generated by dust storms in the Sejzi plain desert area within the industrial district of Isfahan metropolitan, Iran. The enrichment factor revealed the highest values for Zn, Pb, and Cd which among them Zn showed the highest value (8.1) with the potential source of industrial activities confirmed by the integrated pollution index, accumulation coefficient, and ecological risk index. Regarding health risk analysis (non-cancer and cancer risks) the elements including Co, As, and Cr showed a significant risk for adults and children across all seasons. It’s concluded that mitigation of air particles originated from both natural and industrial activities is necessary to reduce their relevant risks to human being and ecosystems in the region.

## Introduction

Dust storms have significantly increased in recent decades, particularly in dry and semi-dry areas, making it impossible to ignore their detrimental effects on air quality and human health^1^. The transmission of these storms occurs through the transportation of essential nutrients, such as iron and phosphorus, to regions that have experienced significant deterioration or have low levels of these nutrients which has subsequently impacted soil components^2,3^. Dust storms often manifest in various regions across the globe and are influenced by diverse meteorological factors. These occurrences are prevalent in predominantly arid and semi-arid regions, encompassing certain nations such as China, United States, Australia, and Africa, as well as numerous western Asian countries, including Iraq, Syria, and Iran^1,^^4–7^. In addition to dust storm origin areas, such as arid and semi-arid regions, its transmission range is very wide and could adversely affect urban and rural areas ^8^. So, it is crucial to identify, analyze, and determine the concentration of dust particles due to their significant impact on air quality, human health, and the environment^9,10^.

Dust particles enter the environment through two ways, weathering of the earth’s crust or through human activities. The issue of pollution caused by potentially toxic elements (PTEs) has emerged as a significant environmental concern in contemporary times. Circular dust particles have been found to be linked to PTEs originating from many sources such as industrial activities, road transport, traffic, dust storms and other natural and industrial resources. The scientific community has long been concerned about the significant impact of dust-related elements on several natural sectors, including air, water, soil, and the biosphere^11–13^. Hence, in numerous studies, experts have suggested other indicators to conduct a more suitable and thorough examination of environmental pollution and health risk associated with PTEs^14,15^. A health risk assessment was undertaken by Taye and Chandra vanshi to evaluate the potential health risks associated with dust in industrial locations^15^. The results of their study indicated that those who were exposed to particles present on road dust through skin contact, oral ingestion, and inhaling might experience various health difficulties. An analysis of cancer and non-cancer risks from several factors in the study revealed that children and elderly individuals had heightened sensitivity to particulate matter, while those who spend extended periods of time on the road face an increased susceptibility^16,17^. In a separate investigation, Liu et al. Their results also showed that the concentration of the elements (e.g. Ni, Cr, Cu, Zn, Cd and Pb) was higher than the background values. The non-carcinogenic risk of each element for children was 10 times higher than for adults. Among these, the non-carcinogenic risk of Cr, Cd, and Pb for children was close to 1 showing the necessity of significant attention^18^. On the other hand, fine particles (e.g. PM_2.5_) that are present in dust have severe consequences for human health. These particles can readily enter the respiratory system and cause a variety of issues due to their small size^19^. These complications include respiratory and cardiovascular disease^20^. Consequently, vulnerable populations, such as the elderly, infants, and those with pre-existing conditions, are at a heightened risk^21^.

To evaluate the potential environmental and health risk associated with the presence of various elements in dust, an analysis was conducted on a range of sources including desert dust storms, industrial activities, road transportation, and heavy traffic. The analysis was performed using indicators of contamination factor (CF), enrichment factor (EF), Integrated Pollution Index (PI), and the Potential Ecological Risk Index (RI). Additionally, estimates for cancer and non-cancer risk were performed for both children and adults. For example, it has been reported that the desiccation of Lake Urmia and the heightened levels of PM_10 _could contribute to elevated health hazards, including higher rates of cardiovascular and respiratory death^17^. The results of a study on the evaluation of carcinogenic and non-carcinogenic risks of edible plant parts affected by PTEs (e.g. Pb, Cu, Fe, Ni, and Zn) showed that the high concentration of Pb in vegetables was concerning, while the risk criterion for other metals was below the permissible limit, and the carcinogenic risk of Ni in women was above the recommended level^22^. Meanwhile, exposure to suspended particles not only increases the risk of diseases but also increases the economic burden^23^. In a study, an increase in Co concentration levels was associated with an increased risk of colorectal cancer^24^. Furthermore, it has been reported that the concentration of As was higher in rural areas, while Cd concentration was higher in urban areas with industrial activities. Health risk assessment showed that children are at greater risk from non-cancer hazards, particularly through ingestion. Lifetime cancer risks (LCR) from exposure to PTEs were observed in adults (LCR = 5.31 × 10^−4^) and children (LCR = 9.05 × 10^−4^). The cancer risk from As was higher in rural areas via ingestion, whereas Ni and Cr risks were higher in urban areas via inhalation and ingestion, respectively. This study estimated that approximately 5 out of 10,000 adults and 9 out of 10,000 children in the study area might develop cancer in their lifetimes due to exposure to indoor PTEs^25^.

Sejzi plain desert, as one of the vital and significant areas in Isfahan province, Iran, holds a great importance in terms of environmental challenges and public health. Additionally, it being an area with extensive industrial activities and road transportation as well as wind erosion, faces more environmental and health issues. Given this situation, a thorough investigation into the ecological and health effects of PTEs associated with dust particles in the region is crucial, as wind erosion, industrial activities, transportation, and road traffic can serve as the main sources of these elements. Therefore, a precise assessment of PTEs and their impacts on human health and the environment in Sejzi plain desert is crucial. Therefore, this study assumes that (1) different seasons of the year have different effects on ecological and health risks of PTEs associated with dust in the industrial desert areas. (2) due to the presence of strong winds in the region, there is a possibility of health and ecosystem risk in affected areas through PTEs associated with dust particles. In fact, assessing ecological risks alongside health risks ultimately leads to decisions regarding the implementation or non-implementation of a particular activity or the use of a specific substance to ensure the health of individuals.

## Materials and methods

### Methodology section

#### Study area

The Sejzi Plain is located between 52°51’ to 52°6’ longitude and 32°38’ to 32°46 latitude, serving as a focal point of wind erosion crises (storms and dust storms), consistently leading to health issues and a decline in the quality of life in Isfahan city. Additionally, the drying of the Gavkhouni International Wetland and the Zayandehrud River due to drought has resulted in increased dust in the Sejzi plain^26,27^.The transportation of dust via air can facilitate the transfer of PTEs from the Sejzi plain to Isfahan city, resulting in an increase in their concentrations. This, in turn, leads to a decline in air quality of the region and has implications for public health^11,28^. Isfahan city, being acknowledged as one of Iran’s more established industrial cities, has had significant advancements in industrial growth within its vicinity, resulting in huge increase of its industrial sectors. As a result, owing to its appeal for migration and its substantial contribution to the nation’s industrial sector, this urban center has emerged as a sought-after location for numerous migrants^29^. I has become one of Iran’s most polluted cities due to the existence of enterprises engaged in iron and steel manufacturing, brick and cement factories as well as oil and petrochemical plants and other industrial plants. Additionally, the city faces significant traffic challenges^10,26,30^. According to the data of Department of Environment and Isfahan Meteorological Centre, the air quality in the city was deemed unhealthy for a total of 86 days over the year of 2013–2014. The occurrence of these hazardous days was predominantly seen during the winter season, primarily due to atmospheric inversion phenomena^9,29^.

#### Sampling and sample preparation

**Fig. 1:**
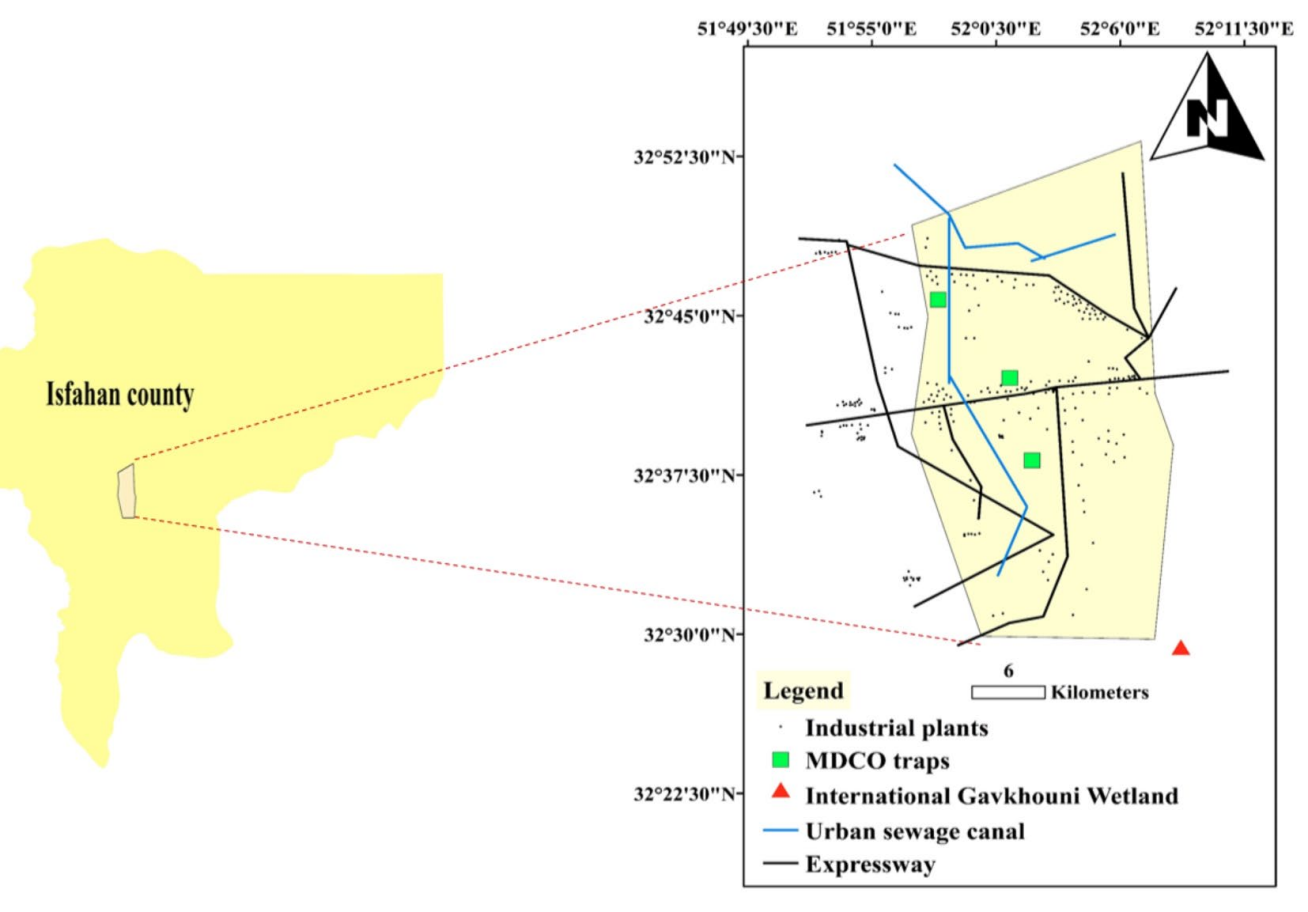
Locations of MDCO traps for collecting dust samples in Sejzi region (Isfahan province, Iran).

Three Marble Dust Collector (MDCO) devices were utilized for this study. The MDCO traps were strategically placed throughout the designated study region (Fig. 1) towards the conclusion of the autumn season in 2023. Subsequently, monthly samples were collected from the traps positioned at the sediment station for a span of one year. Throughout this time frame, the sediments that had accumulated in the traps were gathered by rinsing them with purified water and, once they had dried, were measured using a highly accurate balance with a precision of 0.001 g.

#### Analysis of trace elements with toxicity potential in dust samples

The dust samples were passed through a mesh of 2 mm for the analysis of Co, Mn, V, Ni, Cd, As, Cu, Pb, Cr, Zn, Al, and Ti elements. A sub sample of 2 g of dust particles weighted by a balance with an accuracy of 0.0001 g, transfered into a 50-mL volumetric flask. Then 10 mL of an acid solution (a mixture of concentrated HNO_3_and HCl at a ratio of 1:3) was slowly added to each flask. Subsequently the samples were put on a heater for 1 h at 90 °C, followed by another hour at 120 °C. After cooling, the samples were removed and filled to final volume of 50 mL using distilled water. Finally, the concentration were analyzed using atomic absorption spectrometer (PerkinElmer AAnalyst 700)^31,32^. The blank samples and standard solutions of elements with 3 replications were used to check the quality and accuracy of the results.

#### Statistical analysis

To assess the normality of the data, the Kolmogorov-Smirnov (K-S) test was utilized. Additionally, for data analysis, the one-way analysis of variance (ANOVA) test was employed. Besides, mean comparisons were conducted using the Duncan test at a 95% confidence level. All statistical tests performed using the open-source software R. Moreover, wind rose analysis of the region based on wind speed information from Isfahan Airport station was conducted using the WRPOT (Wind Rose Plot) software^33^.

#### Evaluation of the ecological risk of elements with fluid potential caused by dust

The determination of the EF, Geo-accumulation Index (I geo), Pollution Index (PI), and Potential Ecological Risk Index (PERI) was conducted using the acquired results and the experimental formulas, as presented in Table 1. It should be noted that this study employed data from previous research and global sources in order to establish the reference concentrations of the elements under investigation^34^.

**Table 1:** The ecological risk analysis formulas and pollution level classification^4,14,16,59^.

	Bn	Concentration (mg/kg) background concentration (mg/kg)		
$\:\varvec{E}\varvec{F}=\frac{\left(\frac{\mathbf{C}\mathbf{x}}{\mathbf{A}\mathbf{l}}\right)\mathbf{s}\mathbf{a}\mathbf{m}\mathbf{p}\mathbf{l}\mathbf{e}}{\left(\frac{\mathbf{C}\mathbf{x}}{\mathbf{A}\mathbf{l}}\right)\mathbf{b}\mathbf{a}\mathbf{c}\mathbf{k}\:\mathbf{g}\mathbf{r}\mathbf{o}\mathbf{u}\mathbf{n}\mathbf{d}}$	$\:\mathbf{E}\mathbf{F}$		EF ≤ 2	Low
	Cx sample		2 ≤ EF ≤ 5	Moderate
	Al sample		5 ≤ EF ≤ 20	High
	Cx back ground		20 ≤ EF ≤ 40	Very high
	Al back ground		EF ≥ 40	Extremely
$\:\mathbf{I}\:\varvec{g}\varvec{e}\varvec{o}={\varvec{l}\varvec{o}\varvec{g}}_{2}\left(\frac{\varvec{C}\varvec{i}}{1.5\varvec{B}\varvec{i}}\right)$	Ci	Concentration of HMs in dust samples		Unpolluted to moderately polluted
		(mg/kg),	$$\:0\le\:\text{I}\:\text{g}\text{e}\text{o}\le\:\:1$$	
				Moderately polluted
	Bi	Reference concentration (mg/kg)	$\:2\le\:\text{I}\:\text{g}\text{e}\text{o}\le\:3$	means moderately to strongly Polluted
			$\:3\le\:\text{I}\:\text{g}\text{e}\text{o}\le\:4$	Strongly polluted
			$\:4\le\:\text{I}\:\text{g}\text{e}\text{o}\le\:\:5$	Strongly to very strongly polluted
			$\:\text{I}\:geo\ge\:5$	Extremely polluted environment
Pl *=*$\:\frac{\varvec{C}\varvec{i}}{\varvec{B}\varvec{i}}$	Ci	Concentration of HMs in dust samples		
		(mg/kg),		
	Bi	Reference concentration (mg/kg)		
	PI	Pollution index	PI < 1	Low pollution
			1 < PI < 3	Moderate pollution
			PI > 3	High levels pollution
ERI = Tr×PI	Tr	Concentration of HMs in dust samples		
		Toxic response factor		
	ERI	Ecological risk index	ERI < 40	Low pollution
			40 ≤ ERI < 80	Moderate pollution
			80 ≤ ERI < 160	Higher pollution
			160 ≤ ERI < 320	Serious pollution
			ERI ≥ 320	Sever pollution
	RI	Risk index	RI < 150	Low pollution
RI =∑$\:\frac{\varvec{n}}{1}\:$ERI			150 ≤ RI < 300	Moderate pollution
			300 ≤ RI < 600	High pollution
			RI ≥ 600	Very high pollution

Each element has its own Tr value, which is given as a standard, such that: Co = 5, Mn = 1, V = 2, Ni = 5, Cd = 30, As = 10, As = 10, Cu = 5, Pb = 2, Cr = 2, Zn = 5, Ti = 2 and Al = 2.

Each element has its own Reference concentration value, which is given as a standard, such that Co = 5.38, Mn = 716.00, V = 32.74, Ni = 20.03, Cd = 0.14, As = 5.82, As = 5.82, Cu = 25, Pb = 14.97, Cr = 35.62, Zn = 43.4, Ti = 4010 and Al = 2998.37.

#### Assessment of the health risk of dust elements

The approach employed by the United States Environmental Protection Agency was utilized to evaluate the health risks associated with the constituents found in dust storms and particles released by industries within the designated study region. The primary pathways via which dust enters the human body are skin contact, ingestion, and breathing^35^. The formulation of the detoxification dosage by skin contact, oral intake, and respiration can be determined by employing the equations and parameters indicated in Table 2. The approach employed by the US-EPA was utilized to evaluate the health risks associated with the constituents found in dust storms and particles released by industries within the designated study region. 

**Table 2:** Equations and parameter values of health risk of elements through three exposure routes to dust particles^54,60,61^.

Equation	Pathway	Scale	Definition	Unit	Value	
					Children	Adult
Ding = $\:\mathbf{C}\frac{\mathbf{i}\mathbf{n}\mathbf{h}\mathbf{R}\mathbf{*}\mathbf{E}\mathbf{F}\mathbf{*}\mathbf{E}\mathbf{D}}{\mathbf{B}\mathbf{W}\mathbf{*}\mathbf{A}\mathbf{T}}{\mathbf{CF}}$	Ingestion	C	Concentration	mg/kg	This study	This study
		Ding	Daily dose ingestion	mg/kg day		
		IngR	Ingestion rate	mg/day	200	100
		ED	Exposure duration	Years	24	6
		AT	Averaging time for non-cancer risk	Days	365 × ED	365 × ED
			Averaging time for cancer Risk	Days	65 × 365	65 × 365
		BW	Body weight (site specific)	Kg	75	15
		EF	Exposure frequency (site specific)	Days	180	180
Dinh = $\:\mathbf{C}\frac{\mathbf{i}\mathbf{n}\mathbf{h}\mathbf{R}\mathbf{*}\mathbf{E}\mathbf{F}\mathbf{*}\mathbf{E}\mathbf{D}}{\mathbf{P}\mathbf{E}\mathbf{F}\mathbf{*}\mathbf{B}\mathbf{W}\mathbf{*}\mathbf{A}\mathbf{T}}$		Dinh	Daily dose through inhalation	mg/kg day	20	7.6
Inhalation		InhR	Inhalation rate	m^3^/day		
		PEF	Particle emission factor	mg/m^3^	1.36 × 10^9^	1.36 × 10^9^
Dder = $\:\mathbf{C}\frac{\mathbf{S}\mathbf{L}\mathbf{*}\mathbf{A}\mathbf{S}\mathbf{*}\mathbf{A}\mathbf{B}\mathbf{S}\mathbf{*}\mathbf{E}\mathbf{F}\mathbf{*}\mathbf{E}\mathbf{D}}{\mathbf{B}\mathbf{W}\mathbf{*}\mathbf{A}\mathbf{T}}{\mathbf{CF}}$	Dermal contact	Dder	Daily intake through dermal contact	mg/kg day		
		SA	Exposed skin area	cm^2^	1800	5700
		SL	Skin adherence factor	mg/cm^2^day	0.07	0. 2
		ABS	Dermal adsorption factor	Unitless	0.001	0.001
		CF	Conversion factor	kg/mg	10–6	10–6
LADD = $\:\frac{\mathbf{C}\mathbf{*}\mathbf{E}\mathbf{F}\mathbf{*}\mathbf{E}\mathbf{D}\mathbf{*}\mathbf{C}\mathbf{R}}{\mathbf{B}\mathbf{W}\mathbf{*}\mathbf{A}\mathbf{T}\mathbf{*}\mathbf{P}\mathbf{E}\mathbf{F}}$	All of the three routes	CR	Contact frequency	Mg/kg day	CR = IngR	CR = IngR

AT = EF x ED, ED for adult for cancer risk is 65 year which is the average life expectancy.

Various parameters including standard factor (SF), average daily dosage (D), lifetime average daily dose (LADD) and reference dose (RfD) were used to assess health risks of the PTEs associated with dust particles. These principles demonstrate the calculation of the probability and impact of detrimental effects resulting from exposure to particular elements, as well as the assessment of the level of danger to human health. Health risk (HI) was calculated using Hazard Quotient (HQ) and Lifetime Cancer Risk (LCR) based on the PTEs concentration (Eqs. 1-3)

1 $$\:HQ=\frac{D}{RFD}$$

2 $$\:HI=\sum\:HQ$$

3 $$\:LCR=LADD\times\:SF$$

## Results and discussion

**Fig. 2:**
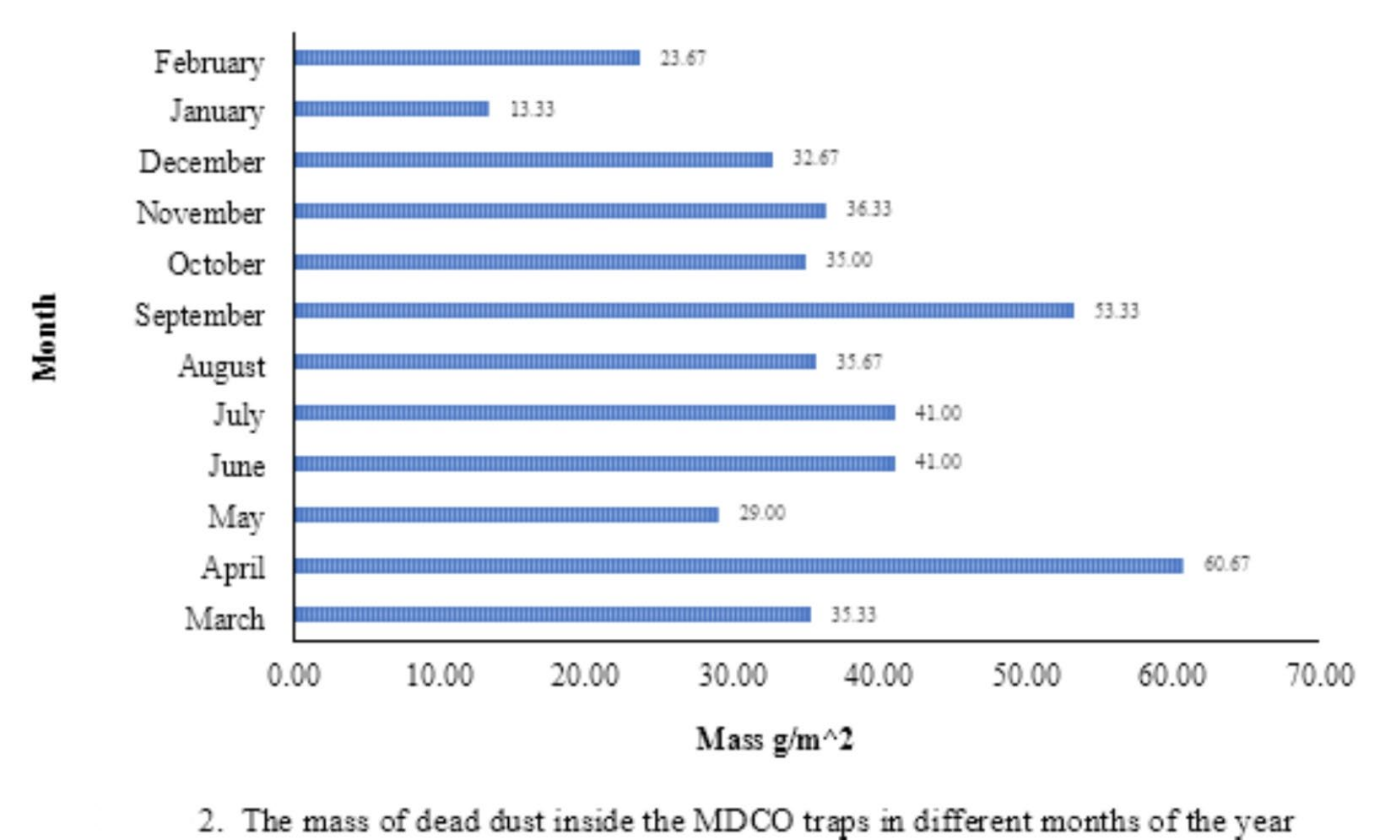
The mass of dead dust inside the MDCO traps in different months of the year.

The data presented in Fig. 2 illustrates the monthly dust deposition rate, which reveals that the most significant deposition rates on the slopes under investigation were observed throughout the spring and summer seasons, specifically in the months of April, September, July, and June. The primary factor contributing to the observed rise in deposition rates throughout this period is the proliferation of dust storms. Based on meteorological data obtained from Isfahan, the aforementioned months have exhibited the most frequent instances of wind erosion events within the region, characterized by velocities beyond the established threshold velocity levels. Furthermore, it is worth noting that the month of January exhibited the lowest rate of dust deposition, measuring at 13.3 g.m^−2^. One of the positive aspects of this research was the comprehensive determination of the health and ecological risk indices in four seasons in the industrial area of Plain Sejzi, as one of the active centers of wind erosion in Iran. However, a significant limitation of this study was the impossibility of sampling every storm and environmental event at the time of occurrence, so samples were collected monthly.

**Fig. 3:**
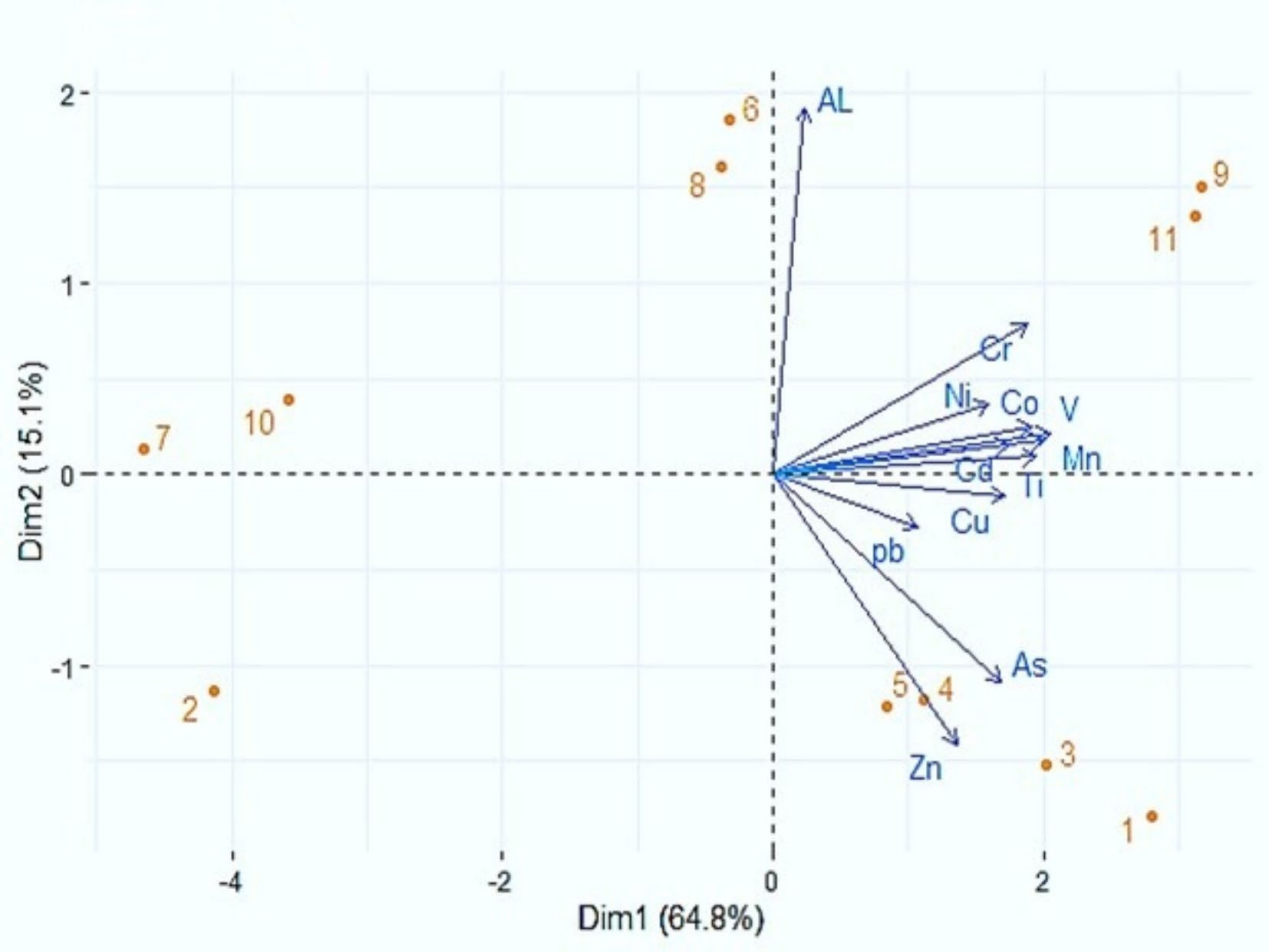
Distribution of the main principal components of elements associated with dust particles in the study area.

**Fig. 4:**
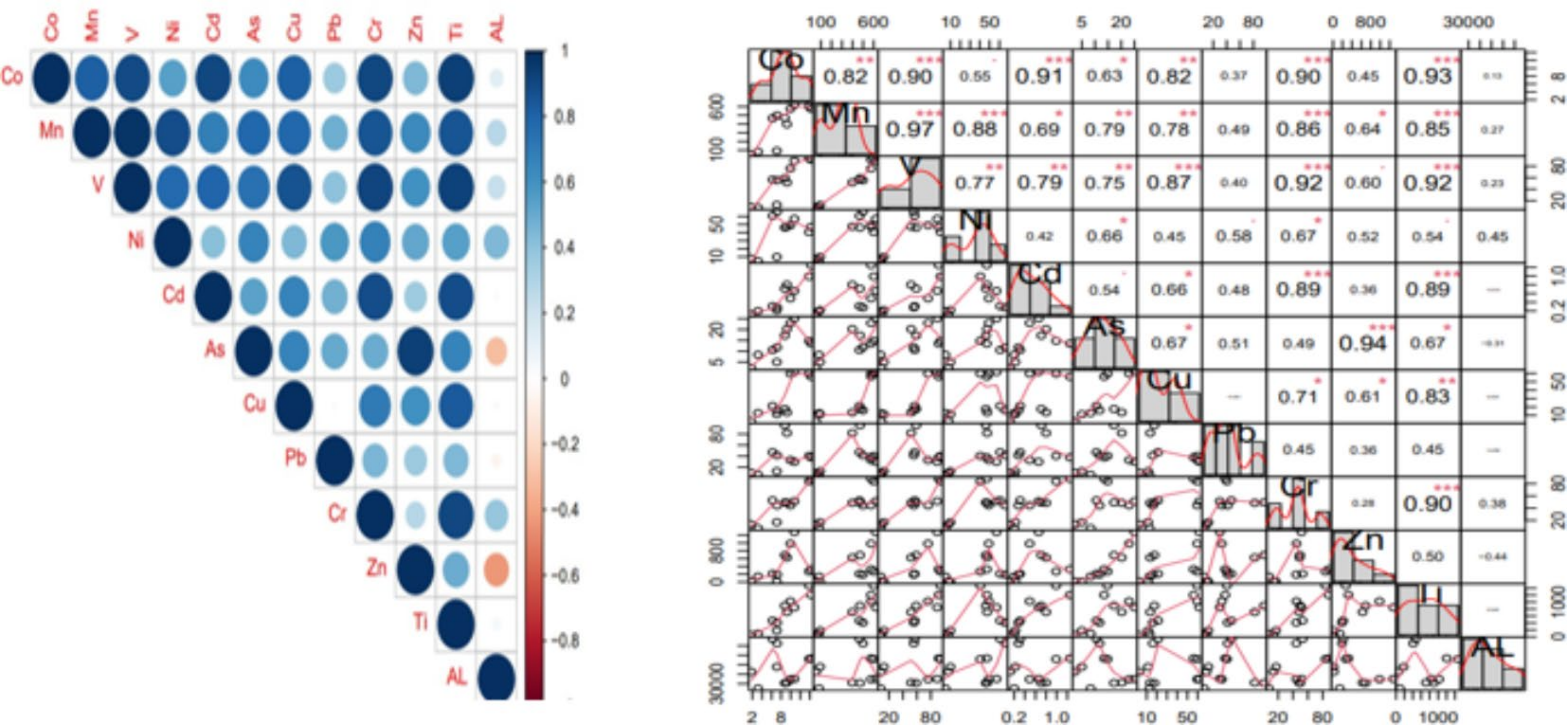
Pearson correlation matrix of the selected elements in the sediment samples from the study.

Table 3 presents a statistical study of the elemental composition of circular dust samples. The results of the statistical analysis indicate that the Al exhibits the highest mean dust element density, whilst the Cd has the lowest mean concentration. A comparative analysis was conducted to assess the relationship between field density and the average elements. It was observed that, apart from Ti, the average density of all the elements examined exceeded the field concentrations, as indicated in Table 4. The K-S test analysis revealed that, except for Cu and Ni, all data, at a significance level of 0.05, conformed to a normal distribution. These results are consistent with studies conducted by^9,11,26,36,37^. The results showed a high level of correlation between the variables (Fig. 3). Additionally, the analysis of PCA results indicated that the first and second components, with eigenvalues of 7.77 and 1.88, respectively, cover 79.20% of the variance, respectively. According to Table 4; Fig. 4, it seems that Zn and As and also Al have a different source compared to the other elements which could have a common source. The sources mentioned may originate from a range of factors, such as emissions from industrial activities, agricultural activities in the plain, the use of petrol and diesel fuels, road transportation, municipal and industrial sewage sludges, brick and gypsum kilns, sand and gravel mines situated within the study area as well as soil erosion. Several causes have the potential to contribute to an elevation in the elemental concentration within the dust of the examined region. Researchers have verified this issue through their research and data analysis^9,16,37–39^.

**Table 3:** The slope factor and reference dose for the three exposure routes^10,55,62–64^.

Metals	RfD _inh_ (mg/kg day)	RfD _der_ (mg/kg day)	RfD _ing_ (mg/ kg) day)	SF_ing_ (mg/kg day)	SF_der_ (mg/kg day)	SF_inh_ (mg/kg day)
Mn	0.000014	0.00183	0.046			
Zn	0	0.075	0.3			
Pb	0	0	0.0036	0	0.042	0.042
Cr	0.00003	0	0.003	0.5	0	41
Cd	0.000057	0.0005	0.0005	0	0	6.3
As	0.0003	0.0003	0.0003	1.5	1.5	15.1
Ni	0	0.0056	0.02	0	0	0
Co	0.0000057	0.0000057	0.02	9.8	0	0

*RfD*_*inh*,_is reference dose for inhalation route,* RfD*_*ing*,_is reference dose for ingestion route,* RfD*_*der*_is reference dose for dermal contact route,* SF*_*inh*_is slop factor for inhalation route,* SF*_*ing*_is slop factor for ingestion route and* SF*_*der*_is slop factor for dermal contact route.

**Table 4:** The total amount of variance (eigenvalue) and variance percent of selected elements in dust samples explained by a given principal component.

	**Eigenvalue**	**Variance (%)**
PCA.1	7.77	64.15
PCA.2	1.88	15.14
	PCA1	PCA.2
Co	0.91	0.12
Mn	0.96	0.08
V	0.98	0.10
Ni	0.76	0.17
Cd	0.84	0.7
As	0.81	−0.52
Cu	0.82	−0.05
Pb	0.51	−0.13
Cr	0.90	0.37
Zn	0.65	−0.67
Ti	0.93	0.45
AL	0.11	0.92

Industrial and manufacturing activity in industrial zones and factory outlets typically lead to an increase in elements. The rise in pollution levels is frequently attributed to industrial activities, including the generation, treatment, and elimination of waste and industrial remnants, which can result in contamination of the air, water, and soil in the region^40^. Furthermore, in settlements located in windy and erosion-prone areas, particles and pollutants are easily transported due to strong winds and soil erosion, resulting in a higher concentration of elements. These factors contribute to an increase in the concentration of elements in these areas, a phenomenon corroborated by recent research studies^41–43^. Data analysis revealed that the concentration of elements in various classes fluctuates, resulting in variations in the average density of elements during the spring, summer, autumn, and winter seasons. For example, the average concentration of Co could be categorized into various groups in the following manner: In winter, the content was 5.04 mg/kg, whereas in other seasons it reached to 7.96 to 11.74 mg/kg. These findings indicated that Co concentrations were roughly 1.3, 1.5, and 2.3 times higher during the spring compared to the autumn, summer, and winter, respectively. Furthermore, the findings from the examination of the other elements indicated that they exhibited varying degrees of concentration across distinct categories. There appear to be notable variations in the concentration of As across different classes.

**Fig. 5:**
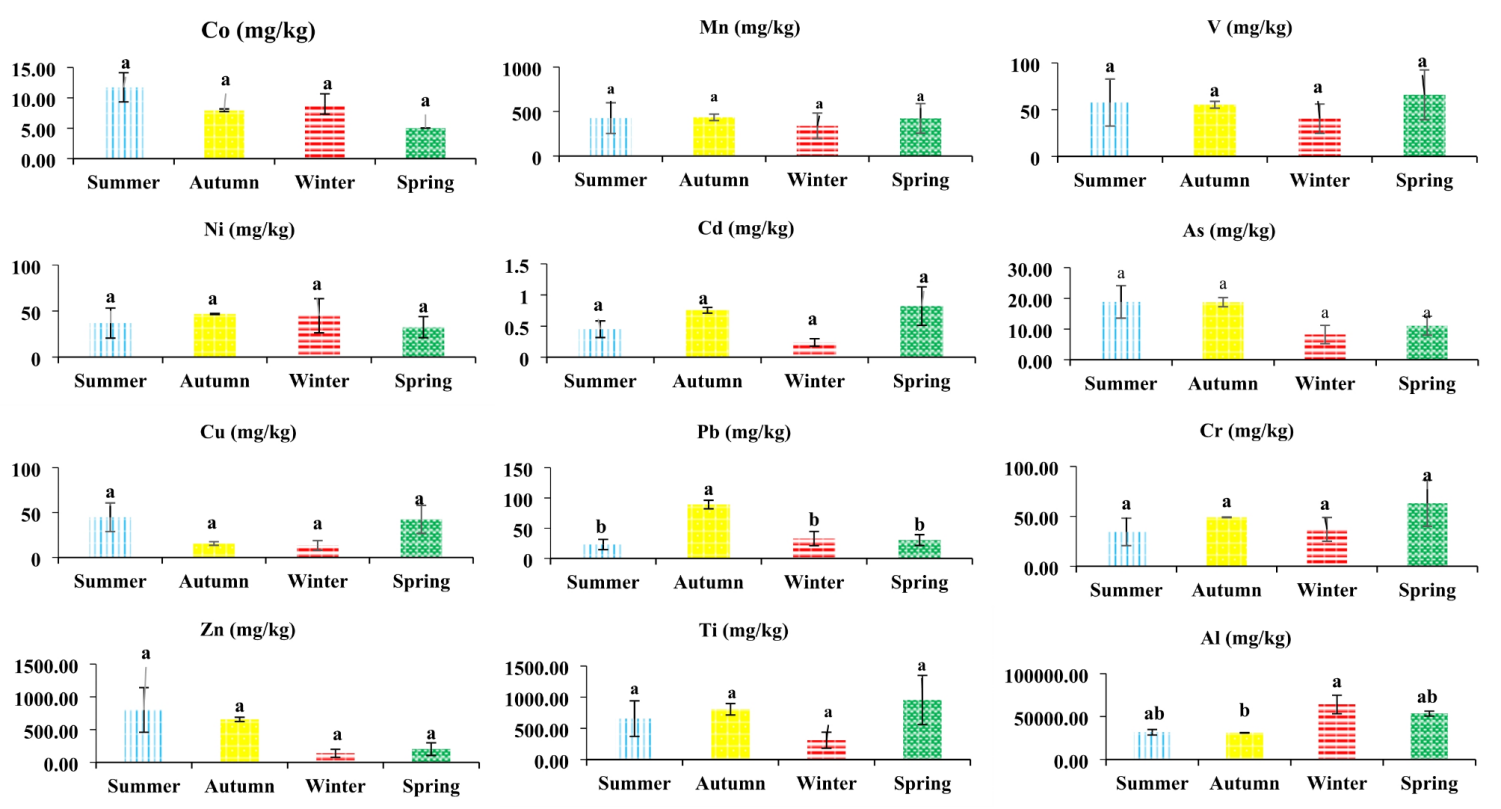
Comparison of average variance analysis between potentially toxic elements and dust over a year.

The variation in the concentration of elements across different seasons could mainly due to changes of activities in potential sources of particles^24^. For examples the natural sources (e.g. wind erosion) are mainly considered in spring and summer, whereas the industrial activities and transportation are the main source throughout a year^22^. Moreover, thermal inversion may promote the accumulation of particles and their associated elements in fall and winter. In the summer season, the concentration of As reaches its peak at 18.82 mg/kg, with the winter season following closely behind at 18.72 mg/kg. Conversely, in the spring and autumn seasons, the As concentrations are comparatively lower, measuring 11.09 mg/kg and 8.20 mg/kg, respectively. It is evident that the concentration of As during the summer is roughly 1.5 times greater than that during the winter. Similarly, during the spring season, the concentration of As is approximately 1.4 times higher than that during the winter season. The findings reveal that the summer season has the highest concentration of Cu at 44.75 m/kg, while the autumn season records the lowest concentration at 16.36 m/kg. Additionally, the results of the analysis of other elements show that the concentration of Pb varies with the season, peaking at 89 mg/kg in the winter and falling to 23 mg/kg in the summer. The observed alterations suggest a decline in lead concentration during the warmer seasons, and an increase during the colder seasons (Table 5; Fig. 5). The levels of chromium (Cr) in dust samples during the spring season (63 mg/kg) exhibit higher values compared to the winter season (49 mg/kg), autumn season (36 mg/kg), and summer season (34 mg/kg). In relation to the concentration of Zn, the examination reveals that it attained its peak and nadir values at 800 and 138 mg/kg, correspondingly, throughout the summer season. Furthermore, the examination of titanium (Ti) concentration throughout several seasons indicates recorded values of 954 mg/kg during the summer season, 805 mg/kg during the winter season, 654 mg/kg during the autumn season, and 309 mg/kg during the spring season, respectively. The analysis of Al concentration ultimately yielded a ranking of Al in terms of magnitude. Winter had a higher concentration of Al (64089 mg/kg) compared to spring (53520 mg/kg), while summer had a higher concentration (31700 mg/kg) than autumn (30987 mg/kg). In winter, the concentration of Al is seen to be 1.19, 2.02, and 2.06 times more than that observed in spring, summer, and fall, respectively (Fig. 5). Environmental experts have identified various factors that contribute to the variations in element concentrations throughout different seasons. These factors include environmental conditions, human activities, and the behavior of chemical elements in the environment^36^.

**Table 5:** The arithmetic mean, maximum, minimum concentrations (mg/kg) and standard errors (SE) of dust samples.

Sampling place		Co	Mn	V	Ni	Cd	As	Cu	Pb	Cr	Zn	Ti	AL
Point 1	Maximum	10.77	621.00	89.14	57.10	0.66	25.10	61.78	32.88	51.40	1287.00	1025.00	35177.00
	Minimum	3.16	82.00	7.90	4.70	0.20	8.36	12.80	6.31	7.30	141.00	92.00	25144.00
	Mean	7.96	426.33	57.68	36.97	0.45	18.82	44.75	23.01	34.43	800.67	654.67	31700.00
	SE	2.41	176.66	25.18	16.30	0.13	5.27	15.99	8.40	13.71	340.65	285.99	3380.17
Point 2	Maximum	9.21	471.00	59.00	47.50	0.80	20.20	17.60	96.00	49.35	689.00	896.00	31198.00
	Minimum	8.77	398.00	51.70	46.20	0.71	17.24	135.50	82.00	48.70	625.00	714.00	30777.00
	Mean	8.99	434.5	55.35	46.85	0.76	18.72	15.55	89.00	49.03	657.00	805.00	30987.50
	SE	0.22	36.5	3.65	0.65	0.05	1.48	2.05	07.00	0.32	32.00	91.00	210.50
Point 3	Maximum	7.16	499.00	56.70	66.00	0.31	11.76	21.20	47.11	52.80	210.00	466.00	77105.00
	Minimum	1.74	53.00	9.10	7.95	0.12	2.29	2.89	9.8	11.69	16.93	52.70	42701.00
	Mean	5.04	339.33	40.45	44.93	0.24	8.20	13.36	32.80	36.95	138.98	309.23	64089.67
	SE	1.67	143.48	15.68	18.55	0.06	2.98	5.45	11.62	11.88	61.30	129.32	10777.96
Total	Maximum	14.27	601.00	93.90	47.1	1.25	14.77	59.66	40.51	88.20	325.00	1477	56778.00
	Minimum	7.10	92.00	12.60	9.86	0.22	5.00	11.32	12.60	17.00	10.60	181.00	47814.00
	Mean	11.74	423.00	65.92	32.50	0.82	11.09	42.38	30.30	63.04	201.87	954.67	53520.67
	SE	2.32	165.66	26.67	11.48	0.31	3.07	15.56	8.88	23.05	96.94	394.66	2826.85

Various studies indicating that the increased activities of industrial communities during periods of higher temperatures may contribute to the escalation of trace metal concentrations, such as Cd, Co, V, Cr, and Ti^44,45^. In addition, the rise in agricultural activities and the application of pesticides for pest management, as well as the improvement of plant resilience and the stimulation of tree development in the periphery of the Sejzi plain, could be additional factors contributing to the increasing concentration of specific elements in this region^26^. Previous research has indicated that the rise in element concentration in the study area could be attributed to the substantial traffic volume and frequent diesel car usage in industrial regions^14,46,47^. In a study examining the ecological and health risks of a region in northwestern China, Zhang and colleagues noted that the use of various pesticides to improve the efficiency of agricultural and livestock production activities may alter the concentration of certain potentially toxic elements in the surrounding soil due to dust sediments collected on the outskirts of farms and agricultural and livestock complexes near the city^48^. Moreover, the study conducted by Nguyen et al. provided evidence that the operations of refineries, factories associated with the steel industry, chemical units, and other industrial facilities have the potential to directly emit pollutants, including Ni, Mn, Pb, and As, into the surrounding environment^49^. Our research reveals that the Sejzi plain, which includes many industrial cities, mines, and brick and gypsum kilns, experiences a rise in the levels of specific components during different seasons. Furthermore, the existence of erosive winds is a key and significant determinant impacting the concentration of elements within this region. According to the research conducted by Ravankhah et al., it was suggested that natural causes could potentially contribute to the transportation and dispersion of pollutants containing dust particles. Robust winds have the ability to convey dust particles containing pollutants from industrial zones to different areas, resulting in a rise in their concentration in the atmosphere^50^. The escalating levels of Ni, Mn, Pb, and As in the dust, which arise from the amalgamation of industrial and natural processes, have led to a surge in air pollution and an escalation in pollutant concentrations within the environment^6,13,51,52^. Moreover, the results of comparing the averages showed that there was no significant difference among the other elements across different seasons of the year, except for Pb and Al (Fig. 5).

The EF findings revealed that the index values for Cd and Zn were 2.8 and 8.1, respectively. Furthermore, the EF index for the other elements under investigation exhibited a diminished level of enrichment. According to the EF, Ni during autumn, Cd during summer and spring, As during summer and winter, and Zn during spring, were categorized as moderately polluted elements. In addition, Pb and Zn were categorized as abundant elements in winter, and both summer and winter, respectively. This might show that for Zn the sources could be active throughout the year and not only because of dust storms which mainly are active in spring and summer in the region. So it mostly comes from industrial and transportation activities in the study area. Regarding the EF, the elements including Ni, Cd, and Zn showed substantial enrichment, whilst the remaining elements demonstrated relatively modest enrichment. Based on a comparative analysis of previous studies and the current research, it is evident that Pb and Zn are commonly found in industrial regions. Soleimani et al. (2018) showed that Pb, Mn, As and Cd had the highest concentration of the selected PTEs in fine particles (i.e. PM_2.5_) in the ambient of Segzi region^53^. The increased concentrations of Cd, Zn, and Ni may be attributed to the influence of human activities on the accumulation of potentially harmful elements in the area, resulting in significant health risk for humans and other living organisms. Methods of transferring potentially hazardous elements, such as Pb, Cu, Zn, Cd, and Ni, encompass the disposal of various vehicle components, including tyres, brake pads, and motor oils. This process results in the release of these elements into the atmosphere, subsequently infiltrating water and soil^33,54,55^. Because Isfahan city is considered as an industrial region and is a crossroad for cargo transportation in Iran, heavy vehicle traffic throughout the year, particularly during summer and winter, contributes to the intense Cd enrichment in this area^40,56^. Regarding the I geo index, the values for the two elements Cd and Zn are 1.05 and 1.73, respectively, placing them in the class of slightly polluted.

The values for other elements were less than 1, placing them in the non-polluted class. The findings from the Igeo index analysis revealed that all elements under investigation fell within the non-contaminated category, apart from Cd during winter and spring, as well as As during winter, which were classified as slightly polluted. It is important to consider the background concentrations of metals in the soil and earth’s crust when computing indices such as the EF and I geo indices^36^. The I geo index analysis revealed that Zn, Cd, and As exhibited positive values, however the remaining elements within this index demonstrated non-polluted characteristics. This could be due to the industrial activities in the region and also the relevant transportation of diesel vehicles and fossil fuels combustion.

**Fig. 6:**
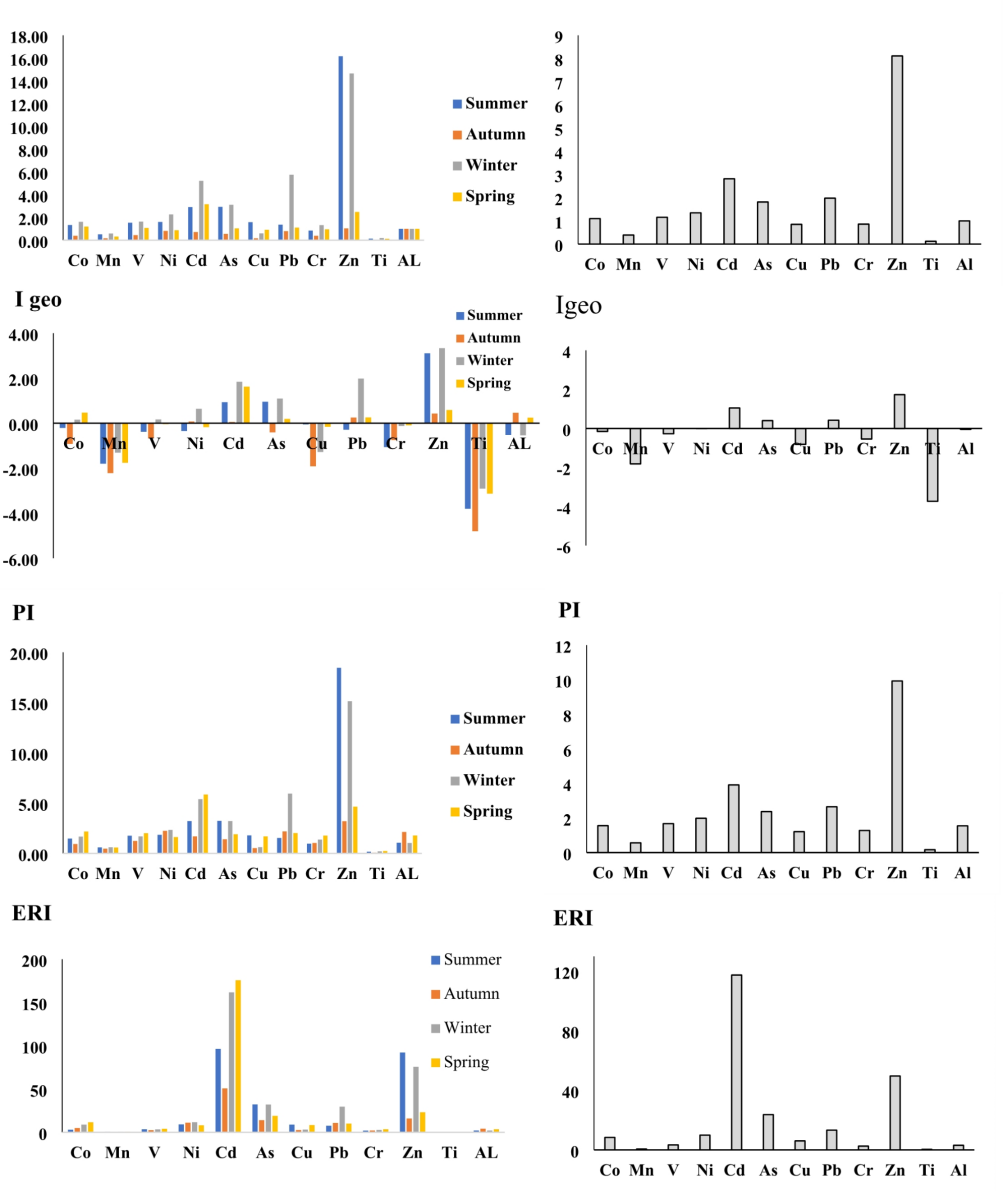
The ecological indices of selected elements in dust particles.

The results regarding the PI index indicated that Zn and Cd were in the extremely high class, while other elements except for Mn and Ti were in the moderate class. Additionally, it showed that among the elements, only Cr and Ti were in the low class in all seasons, and for the other elements, as well as lead and cadmium in all seasons in the high class, lead in winter, and arsenic in summer and winter, they were in the high class. Regarding the ERI index, the findings indicated that during the summer season, 88.33% of the samples fell into the low category, while 16.66% were classified as high. During the autumn season, 91.61% of individuals were classified as low class, while 8.33% were classified as middle class. In the winter season, the research revealed that 88.33% of the samples fell into the low category, while 8.33% were classified as moderate and 8.33% as serious. During the spring season, 91.66% of the samples fell into the low category, while 8.33% were classified as serious Fig. 6. These findings suggested that the elevated levels of Cd and Pb in the study area could be attributed to the combustion of fossil fuels which has been reported previously^9^. In addition, the results of the RI index revealed a value of 238 for the given area, suggesting a classification of a moderate ecological risk.

Additionally, this research conducted a comprehensive examination of the health risk, both non-carcinogenic and carcinogenic, linked to the constituents found in dust storms coming from industrialized urban areas. A comprehensive examination of the correlation between the values of the HI for different elements and various age cohorts reveals that this index can be a valuable instrument for evaluating non-carcinogenic health risk linked to exposure to diverse mineral and metallic elements, particularly across various environmental conditions and seasons. Conversely, a comprehensive examination of these data pertaining to each age cohort unveils noteworthy observations. This investigation reveals those numerous elements, such as Co, Ni, Cd, As, Pb, Cr, Zn, Mn, and V, exhibit HI index values over 1 in numerous instances within the child age range.

This somewhat indicates an increased likelihood of non-cancerous diseases occurring in this age group. Additionally, in some cases, the HI values for children exceed those of adults. The analysis of the HI index results for cobalt shows values exceeding 1 for both children and adults. Furthermore, ingesting this element poses a lower risk compared to the other two methods. Moreover, the HI values for this element exhibit a seasonal trend of spring > autumn > summer > winter in both age groups of children and adults. For the Ni, the results indicated that, except for the autumn season, it has a safe and non-hazardous level for children in all seasons, for both age groups. The HI index for both age groups of children and adults in relation to the element cadmium indicated that for the children’s age group, during the spring and autumn seasons, the HI index exceeds 1, indicating an unsafe and hazardous level.

This also increases the likelihood of non-cancerous diseases. However, for the adult age group, the HI index is less than 1 for all seasons. Furthermore, facing the risk for this element showed a consistent trend through CDI ingest, CDI inhale, and CDI dermal. The analysis of non-carcinogenic risk results for as indicates that it only has a safe and non-hazardous level for the adult age group. Regarding Cu, it also has a safe and non-hazardous level for both age groups of children and adults. However, the analysis of the HI index values for Pb (in autumn), Cr (spring > autumn > winter), and Zn also showed HI values exceeding 1 during the summer season for children (Table 6).

**Table 6:** Non-carcinogenic risk for different exposure pathways to dust particles for children and adults in the study area.

		Summer	Autumn	Winter	Spring	Summer	Autumn	Winter	Spring
	Mean	Child	Child	Child	Child	Adult	Adult	Adult	Adult
Co	HQ ingest	0.04	0.04	0.02	0.06	0.00	0.00	0.00	0.00
	HQ inhale	139.85	141.72	78.21	206.28	0.99	1.00	0.55	1.46
	HQ dermal	139.85	141.72	78.21	206.28	0.99	1.00	0.55	1.46
	HI	279.73	283.48	156.45	412.62	1.98	2.00	1.10	2.91
Ni	HQ ingest	0.19	0.26	0.19	0.16	0.00	0.00	0.00	0.00
	HQ inhale	0.00	0.00	0.00	0.00	0.00	0.00	0.00	0.00
	HQ dermal	0.66	0.92	0.66	0.58	0.00	0.01	0.00	0.00
	HI	0.85	1.18	0.85	0.74	0.01	0.01	0.01	0.01
Cd	HQ ingest	0.09	0.12	0.07	0.16	0.00	0.00	0.00	0.00
	HQ inhale	0.79	1.07	0.59	1.44	0.01	0.01	0.00	0.01
	HQ dermal	0.09	0.12	0.07	0.16	0.00	0.00	0.00	0.00
	HI	0.97	1.31	0.72	1.77	0.01	0.01	0.01	0.01
As	HQ ingest	6.28	5.34	2.35	3.70	0.04	0.04	0.02	0.03
	HQ inhale	6.28	5.34	2.35	3.70	0.04	0.04	0.02	0.03
	HQ dermal	6.28	5.34	2.35	3.70	0.04	0.04	0.02	0.03
	HI	18.85	16.03	7.04	11.11	0.13	0.11	0.05	0.08
Cu	HQ ingest	0.12	0.05	0.03	0.11	0.00	0.00	0.00	0.00
	HQ inhale	0.00	0.00	0.00	0.00	0.00	0.00	0.00	0.00
	HQ dermal	0.19	0.07	0.04	0.18	0.00	0.00	0.00	0.00
	HI	0.31	0.12	0.06	0.29	0.00	0.00	0.00	0.00
Pb	HQ ingest	0.64	2.09	0.71	0.84	0.00	0.01	0.01	0.01
	HQ inhale	0.00	0.00	0.00	0.00	0.00	0.00	0.00	0.00
	HQ dermal	0.00	0.00	0.00	0.00	0.00	0.00	0.00	0.00
	HI	0.64	2.09	0.71	0.84	0.00	0.01	0.01	0.01
Cr	HQ ingest	1.15	1.68	0.97	2.11	0.01	0.01	0.01	0.01
	HQ inhale	114.99	167.92	96.93	210.51	0.81	1.19	0.68	1.49
	HQ dermal	0.00	0.00	0.00	0.00	0.00	0.00	0.00	0.00
	HI	116.14	169.60	97.90	212.61	0.82	1.20	0.69	1.50
Zn	HQ ingest	0.27	0.17	0.03	0.07	0.00	0.00	0.00	0.00
	HQ inhale	0.00	0.00	0.00	0.00	0.00	0.00	0.00	0.00
	HQ dermal	1.07	0.68	0.14	0.27	0.01	0.00	0.00	0.00
	HI	1.34	0.85	0.17	0.34	0.01	0.01	0.00	0.00
Mn	HQ ingest	0.93	0.97	0.60	0.92	0.01	0.01	0.00	0.01
	HQ inhale	3050.80	3184.38	1975.03	3026.95	21.54	22.49	13.95	21.37
	HQ dermal	23.34	24.36	15.11	23.16	0.16	0.17	0.11	0.16
	HI	3075.07	3209.71	1990.74	3051.03	21.71	22.66	14.06	21.54
V	HQ ingest	0.83	0.80	0.46	0.94	0.01	0.01	0.00	0.01
	HQ inhale	0.83	0.80	0.46	0.94	0.01	0.01	0.00	0.01
	HQ dermal	82.55	79.86	46.27	94.34	0.58	0.56	0.33	0.67
	HI	84.20	81.46	47.20	96.23	0.59	0.58	0.33	0.68

The obtained values indicate that the carcinogenic risk for many elements has increased in most samples and, in some cases, has exceeded the permissible limit. This study has focused on the results of risk assessment and safety in both children and adults. The values obtained have revealed that certain samples pose a higher level of hazard and risk for both children and adults. The analysis of carcinogenic risk results for the element Co for both children and adults showed that it is ($\:1\times\:{10}^{-6}\:$to $\:1\times\:{10}^{-4}$) for all seasons, indicating that they are under control and surveillance. Similarly, for Ni and Cd, the data are less than ($\:1\times\:{10}^{-6}\:$ ); this is a negligible condition for both children and adults. The analysis of the results for the concentration of arsenic (As) showed that the carcinogenic risk of this element for the children age group during summer, autumn, and spring is in the hazardous class ($\:1\times\:{10}^{-4}$), and for autumn, it falls into the negligible class. For adults, in all seasons, the situation is under control and surveillance (ranging from $\:1\times\:{10}^{-6}$ to $\:1\times\:{10}^{-4}$). Similarly, the carcinogenic risk status for Pb was such that during the summer, winter, and spring seasons, it was under control and surveillance (ranging from $\:1\times\:{10}^{-6}$ to $\:1\times\:{10}^{-4}$), while for the autumn season, it was less than (below $\:1\times\:{10}^{-6}$). However, the area’s high chromium concentration classified both children and adults as having a carcinogenic health risk (greater than $\:1\times\:{10}^{-4}$). Moreover, the examination of the findings pertaining to the element Zn revealed that the target carcinogenic risk (TCR) was found to be below ($\:1\times\:{10}^{-6}$) for both the paediatric and adult age cohorts. According to Table 7, the results indicate that elements Al, Zn, and Ti have the highest impact on carcinogenic risk. Therefore, contact pathways through ingestion and inhalation showed the highest and lowest non-carcinogenic risks. Wang’s study validated this assertion^57^. Due to their extremely low carcinogenic risk, people typically overlook the elements Mn, V, Ti, and Al^10,58^.

**Table 7:** Carcinogenic risk for different exposure pathways to dust particles for children and adults in the study area.

		Summer	Autumn	Winter	Spring	Summer	Autumn	Winter	Spring
	Mean	Child	Child	Child	Child	Adult	Adult	Adult	Adult
Co	CR ingest	0.00	0.00	0.00	0.00	0.00	0.00	0.00	0.00
	CR inhale	4.40E-05	2.79E-05	4.97E-05	6.49E-05	1.89E-05	1.20E-05	2.14E-05	2.79E-05
	CR dermal	0.00	0.00	0.00	0.00	0.00	0.00	0.00	0.00
	TCR	4.40E-05	2.79E-05	4.97E-05	6.49E-05	1.89E-05	1.20E-05	2.14E-05	2.79E-05
Ni	CR ingest	0.00	0.00	0.00	0.00	0.00	0.00	0.00	0.00
	CR inhale	0.00	0.00	0.00	0.00	0.00	0.00	0.00	0.00
	CR dermal	0.00	0.00	0.00	0.00	0.00	0.00	0.00	0.00
	TCR	0.00	0.00	0.00	0.00	0.00	0.00	0.00	0.00
Cd	CR ingest	0.00	0.00	0.00	0.00	0.00	0.00	0.00	0.00
	CR inhale	1.60E-06	8.42E-07	2.69E-06	2.92E-06	6.88E-07	3.62E-07	1.15E-06	1.25E-06
	CR dermal	0.00	0.00	0.00	0.00	0.00	0.00	0.00	0.00
	TCR	1.60E-06	8.42E-07	2.69E-06	2.92E-06	6.88E-07	3.62E-07	1.15E-06	1.25E-06
As	CR ingest	1.59E-05	6.94E-06	1.59E-05	9.39E-06	6.85E-06	2.98E-06	6.81E-06	4.03E-06
	CR inhale	1.60E-04	6.99E-05	1.60E-04	9.46E-05	6.89E-05	3.00E-05	6.86E-05	4.06E-05
	CR dermal	1.59E-05	6.94E-06	1.59E-05	9.39E-06	6.85E-06	2.98E-06	6.81E-06	4.03E-06
	TCR	1.92E-04	8.38E-05	1.91E-04	1.13E-04	8.26E-05	3.60E-05	8.22E-05	4.87E-05
Cu	CR ingest	0.00	0.00	0.00	0.00	0.00	0.00	0.00	0.00
	CR inhale	0.00	0.00	0.00	0.00	0.00	0.00	0.00	0.00
	CR dermal	0.00	0.00	0.00	0.00	0.00	0.00	0.00	0.00
	TCR	0.00	0.00	0.00	0.00	0.00	0.00	0.00	0.00
Pb	CR ingest	1.10E-07	1.57E-07	4.27E-07	1.45E-07	4.74E-08	6.76E-08	1.83E-07	6.25E-08
	CR inhale	5.46E-07	7.78E-07	2.11E-06	7.19E-07	2.34E-07	3.34E-07	9.07E-07	3.09E-07
	CR dermal	0.00	0.00	0.00	0.00	0.00	0.00	0.00	0.00
	TCR	6.56E-07	9.35E-07	2.54E-06	8.64E-07	2.82E-07	4.02E-07	1.09E-06	3.71E-07
Cr	CR ingest	9.72E-06	1.04E-05	1.38E-05	1.78E-05	4.18E-06	4.48E-06	5.95E-06	7.64E-06
	CR inhale	7.97E-04	8.55E-04	1.13E-03	1.46E-03	3.42E-04	3.67E-04	4.88E-04	6.27E-04
	CR dermal	0.00	0.00	0.00	0.00	0.00	0.00	0.00	0.00
	TCR	8.07E-04	8.66E-04	1.15E-03	1.48E-03	3.47E-04	3.72E-04	4.93E-04	6.34E-04
Zn	CR ingest	0.00	0.00	0.00	0.00	0.00	0.00	0.00	0.00
	CR inhale	0.00	0.00	0.00	0.00	0.00	0.00	0.00	0.00
	CR dermal	0.00	0.00	0.00	0.00	0.00	0.00	0.00	0.00
	TCR	0.00	0.00	0.00	0.00	0.00	0.00	0.00	0.00

## Conclusion

This study reveals spatiotemporal variations of PTEs concentration associated with dust in the Sejzi region as an industrial desert area. Increase of PTEs concentration in spring and summer could be attributed to a multitude of sources, encompassing industrial operations, agricultural practices, and natural sources such as soil erosion and dust storms. Through the examination of indices such as EF, I geo, and PI, the findings suggested that elements such as Zn, Cd, and Pb demonstrated the highest concentration and enrichment factor in comparison to other elements. It seems that the main sources of these elements, particularly Zn are industrial activities and the relevant transportation in the region, while the other elements could mainly origin from natural sources. Based on the RI index, the region is categorized as having a moderate level of ecological risk of PTEs. The analysis of health indices revealed that both children and adults were exposure to the risk of the pollutants, particularly the former group. Moreover, the acquired data suggested that the carcinogenic hazard had escalated for numerous elements and, in certain instances, has surpassed acceptable thresholds. This study aims to determine the environmental health condition of individuals who have been exposed to PTEs. It highlights the significance and need for implementing suitable steps to mitigate the relevant risks in the region. This highlights the necessity of implementing effective strategies and measures to regulate and alleviate environmental pollution in this region, thereby safeguarding human well-being and promoting environmental conservation.

## Data Availability

The datasets used and/or analyzed during the current study available from the corresponding author on reasonable request.
